# Evaluating attention deficit hyperactivity disorder symptoms in children and adolescents through tracked head movements in a virtual reality classroom: The effect of social cues with different sensory modalities

**DOI:** 10.3389/fnhum.2022.943478

**Published:** 2022-08-04

**Authors:** Yoon Jae Cho, Jung Yon Yum, Kwanguk Kim, Bokyoung Shin, Hyojung Eom, Yeon-ju Hong, Jiwoong Heo, Jae-jin Kim, Hye Sun Lee, Eunjoo Kim

**Affiliations:** ^1^Department of Psychiatry, Yonsei University College of Medicine, Seoul, South Korea; ^2^Department of Neurology, Yonsei University College of Medicine, Seoul, South Korea; ^3^Department of Computer Science, Hanyang University, Seoul, South Korea; ^4^Institute of Behavioral Science in Medicine, Yonsei University College of Medicine, Seoul, South Korea; ^5^Biostatistics Collaboration Unit, Department of Research Affairs, Yonsei University College of Medicine, Seoul, South Korea; ^6^Department of Psychiatry, Yonsei University College of Medicine, Gangnam Severance Hospital, Seoul, South Korea

**Keywords:** head movement, ADHD, virtual reality, social cues, multiple sensory modalities

## Abstract

**Background:**

Attention deficit hyperactivity disorder (ADHD) is clinically diagnosed; however, quantitative analysis to statistically analyze the symptom severity of children with ADHD via the measurement of head movement is still in progress. Studies focusing on the cues that may influence the attention of children with ADHD in classroom settings, where children spend a considerable amount of time, are relatively scarce. Virtual reality allows real-life simulation of classroom environments and thus provides an opportunity to test a range of theories in a naturalistic and controlled manner. The objective of this study was to investigate the correlation between participants’ head movements and their reports of inattention and hyperactivity, and to investigate how their head movements are affected by different social cues of different sensory modalities.

**Methods:**

Thirty-seven children and adolescents with (*n* = 20) and without (*n* = 17) ADHD were recruited for this study. All participants were assessed for diagnoses, clinical symptoms, and self-reported symptoms. A virtual reality-continuous performance test (VR-CPT) was conducted under four conditions: (1) control, (2) no-cue, (3) visual cue, and (4) visual/audio cue. A quantitativecomparison of the participants’ head movements was conducted in three dimensions (pitch [head nods], yaw [head turns], and roll [lateral head inclinations]) using a head-mounted display (HMD) in a VR classroom environment. Task-irrelevant head movements were analyzed separately, considering the dimension of movement needed to perform the VR-CPT.

**Results:**

The magnitude of head movement, especially task-irrelevant head movement, significantly correlated with the current standard of clinical assessment in the ADHD group. Regarding the four conditions, head movement showed changes according to the complexity of social cues in both the ADHD and healthy control (HC) groups.

**Conclusion:**

Children and adolescents with ADHD showed decreasing task-irrelevant movements in the presence of social stimuli toward the intended orientation. As a proof-of-concept study, this study preliminarily identifies the potential of VR as a tool to understand and investigate the classroom behavior of children with ADHD in a controlled, systematic manner.

## Introduction

Attention deficit hyperactivity disorder (ADHD) is a common neurodevelopmental disorder characterized by inattention, hyperactivity, and impulsivity, observed in multiple daily environments (American Psychiatric Association, 2013). ADHD is associated with impairments in academic and social functioning, and if not treated properly, can be accompanied by a range of comorbid psychiatric disorders (Biederman et al., 1991; Biederman et al., 2004; Spencer et al., 2007). The current gold standard for the clinical assessment of children and adolescents with ADHD is mainly based on subjective reports from the patients themselves or their caregivers and teachers about their behavior at home and school. Such reports may be inaccurate or biased, and a lack of understanding of ADHD students’ behavior in a classroom environment may lead to more inaccurate evaluations. Thus, the lack of an objective means of evaluating ADHD is a significant drawback.

Efforts have been made to incorporate objective neuropsychological tests, such as the continuous performance test (CPT), to evaluate sustained attention (Corkum and Siegel, 1993; Hall et al., 2016; Bloch et al., 2017). In particular, virtual reality CPT (VR-CPT) has been widely used in the field of ADHD for the assessment, treatment, and rehabilitation of patients (Pollak et al., 2010; Parsons et al., 2017). Previous studies have reported that VR tasks used for patients with ADHD better reflect the participants’ performance in daily life compared to traditional computerized neuropsychological tests, suggesting the potential use of VR technology for the objective assessment of attentional symptoms (Rizzo et al., 1999; Rizzo et al., 2004; Rizzo et al., 2006; Parsons, 2015; Bashiri et al., 2017). However, prior attempts to assess ADHD symptoms based on CPTs have mostly focused on the attention and impulsivity aspects of the disorder, with limited assessment of the hyperactivity and motor behavior components (Teicher et al., 2008; Ohashi et al., 2010).

The US FDA has started to focus on real-world data from novel digital sources/monitoring and neurobehavioral testing, suggesting a trend toward emphasizing the role of technology in assisting and augmenting clinical care (Keshav et al., 2019). Furthermore, the development of technologies that can quantify behavior provides us with a means to use them to retrieve patient-generated data in VR settings. In this study, we utilized a head-mounted display (HMD) to automatically track and record real-time body movements of participants in the virtual world. Several studies have used head movement to represent hyperactivity in individuals with ADHD (Hong et al., 2021). Previous research has shown that head movement toward target stimuli is an especially important indicator of attentional orientation, suggesting that further research on this is warranted (Teicher et al., 1996; Lavine et al., 2002; Seyama, 2006; Frischen et al., 2007). Our approach includes these objective locomotor measures provided by the HMD, allowing a more comprehensive approach to the assessment of ADHD compared to most CPT-based studies. Although capturing the movement itself is not clinically valid or useful, it certainly opens new fields for the study and validation of this approach.

While the coronavirus disease (COVID-19) pandemic has triggered research on the behavior of students in virtual environments for the general pediatric population, there have been few studies on children and adolescents with ADHD. This field of study is critical in the current situation, in which a large proportion of education is being replaced with remote online learning. It is important to understand how a teacher giving instructions on the screen influences attention performance in both individuals with and without ADHD and how they differ in an online classroom environment. VR technology has the advantage that it can be used to construct desired environments, including various stimuli that may influence attention performance. Thus, it provides the participant with a more ecologically valid environment for assessment compared to the conventional CPT conducted in a quiet, isolated room. In addition, these environments and stimuli can be easily manipulated and fully controlled by the experimenter, allowing assessment of the impact of each stimulus on patients’ behavior (Hong et al., 2021).

Generally, children are required to utilize their attention in the presence of various environmental stimuli and interactions with teachers. This study focused on how specific features of a VR program may affect the attention performance of patients with ADHD. For example, the wide variety of contexts that can be simulated within VR allows us to compare the effects of different degrees of social cues or stimuli of different sensory modalities in a differential manner. We implemented the social component by adding a teacher avatar that could move and vocalize to mediate the attention of participants in the VR classroom setting. Previous studies have shown that gestures or gaze cues from a teacher in a VR classroom environment can evoke attention shifts, leading to similar motor responses in the student, such as gaze and head movements (Mansfield et al., 2003; Bayliss et al., 2004; Seyama, 2006). Attention has also been shown to improve in the presence of social cues (e.g., different social distances, gestures, and verbal instructions) from the avatar teacher (Eom et al., 2019). These results suggest that social factors may significantly influence attention. If social cues are found to have effect of the attention deficit of ADHD individuals, training involving enhancement in perceiving social stimuli could be implemented to help individuals to perform with better attention in real-life situations.

To implement stimuli with multiple sensory modalities, we created several conditions for the participants to perform the evaluation, each with a stepwise increase in the presentation of sensory modalities. Prior studies suggest that the hyperactivity domain is associated with the sensory modality of the stimulus (e.g., visual or auditory) and that it may be differentially affected in each modality in individuals with ADHD (Schmidt et al., 2019). Investigation of the effect of multisensory stimuli in the sustained attention of ADHD individuals may provide direction for designing new intervention content specifically tailored and adjusted to be more efficient for students with ADHD. For example, if ADHD individuals are found to function better in the presence of multisensory cues in assisting attention, virtual or online tools for their education may be designed to integrate this aspect. On the other hand, if they are found to have difficulties in utilizing cues of multiple sensory modalities, it may be more beneficial to help ADHD students concentrate by using simple cues from a single sensory modality.

Based on this background, head-movement data based on a head-tracking unit on an HMD device were collected by our VR system, and its potential as an objective index of hyperactivity was examined. We also investigated how specific features in a VR classroom environment influence the attention of children and adolescents. While most previous studies on VR classrooms have focused on an individual’s regulation, control, and management of cognitive processes, we created a VR environment in which a virtual teacher alerts the participants toward the stimuli of the VR-CPT via visual (gesturing) and/or verbal instructions. In this proof-of-concept study, we hypothesized the following: First, head movement will show a significant correlation with the current standard clinical symptom measurements, and thus, would be a valid digital phenotype that may represent ADHD severity objectively. Second, differently simulated social or sensory cues from a teacher avatar will affect head movement in both ADHD and healthy control (HC) participants in the VR classroom environment. Finally, children with and without ADHD will exhibit different patterns in response to various cues simulated in our VR setting. We believe that if particular cognitive or behavioral profiles under different stimuli conditions in ADHD and HC groups can be established through our study, our VR system may serve as a useful tool for clinicians to understand the characteristics of patients with ADHD in greater depth in terms of their responses to environmental stimuli.

## Materials and methods

### Participants and procedures

Twenty children and adolescents with ADHD and 17 HCs participated in this study. The inclusion criteria were as follows: (1) ADHD diagnosis according to the DSM-V criteria, (2) age of 6–17 years, and (3) allowance of common comorbid disorders, such as depressive disorder, anxiety disorder, and oppositional defiant disorder. The exclusion criteria were as follows: (1) an intelligence quotient (IQ) < 70, (2) past or current serious medical or neurological diseases, and (3) comorbid autism spectrum disorder, psychotic spectrum disorder, or bipolar disorder.

All participants with ADHD were taking medications, such as stimulants for ADHD symptoms, aripiprazole for irritability, and/or escitalopram for depression or anxiety. As methylphenidates can affect the performance of attentional tasks, those taking methylphenidates were required to stop medication for at least 48 h before participating in the study. Psychiatric interviews with HCs were also conducted to rule out any past or current medical and/or psychiatric disorders, and psychotropic medication usage.

All participants underwent a practice session to familiarize themselves with the VR-CPT. Following the practice session, the participants were presented with the four VR conditions for the task. The order of the VR-CPT conditions was counterbalanced to control for systematic order effects. After going through all four VR conditions, the participants filled out a debriefing questionnaire regarding their VR experience. This study was approved by the Institutional Review Board of Yonsei University College of Medicine, Gangnam Severance Hospital, and informed consent was obtained from all participants and their guardians prior to participation.

### Materials

#### Diagnostic procedures and assessment of clinical symptoms

The diagnosis of ADHD and comorbid disorders was established based on the Mini-International Neuropsychiatric Interview for Children and Adolescents (Version 6.0; Sheehan et al., 1998; Sheehan et al., 2010) for both ADHD and HC participants. IQs were obtained using the short form of the Korean version of the Wechsler Intelligence Scale for Children, Third Edition (K-WISC III; Kwak et al., 2002).

#### Attention deficit hyperactivity disorder rating scale–IV, Korean Version (ADHD-RS-IV)

The severity of ADHD symptoms was assessed using the Korean version of the ADHD Rating Scale (ADHD-RS) IV (So et al., 2002). This 18-item Likert scale was filled out by parents, who rated the symptom criteria of their children with ADHD on a severity scale ranging from 0 (not present) to 3 (severe). Higher scores on the ADHD-RS indicate higher severity of symptoms, such as inattention, impulsivity, and hyperactivity.

#### Korean child behavior checklist

The child behavior checklist (CBCL) is an assessment tool completed by parents to measure the behavioral problems of children. The Korean CBCL (K-CBCL) is a standardized and validated instrument used to evaluate the behavior of children aged 6–18 years [Cronbach’s α = 0.62–0.95, (Oh et al., 1997; Achenbach and Ruffle, 2000; Lee et al., 2015)]. The K-CBCL comprises 119 items, which provide a raw score and normalized T-score based on sex and age (*M* = 50, SD = 10), with higher T-scores indicating more problematic behavior.

#### Virtual reality continuous performance test

We developed a system that captures and quantifies the motor activity of participants while they are engaged in a CPT task. The virtual reality continuous performance test (VR-CPT) utilized was based on the two-button AX-CPT version of the CPT (Eom et al., 2019), which lasted 15 min. CPT attention measures and the degree of head motion of each participant were automatically recorded and calculated using a VR system. In this task, the participants were required to press the right arrow on the keyboard as fast as they could upon the appearance of an X after an A. In response to stimuli presented otherwise, the participants were required to press the left arrow key. A total of 24 target stimuli, each with a duration of 100 ms, were presented. Interstimulus intervals varied from 1,500 to 2,500 ms.

#### Task conditions

**FIGURE 1:**
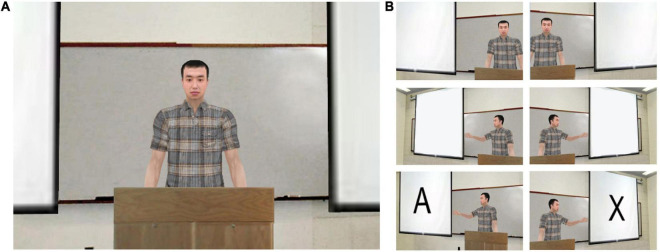
Virtual reality (VR) classroom environment. VR classroom condition presented to the participants. **(A)** No-cue condition, in which the teacher avatar was presented in the center of the visual field without presenting any social cues. **(B)** The visual cue (hand gesture) provided by the teacher avatar in the visual cue condition and the visual/audio cue condition. The images are presented in chronological order; the teacher avatar gestures toward one screen, then the stimulus appears on the pointed screen.

We constructed four experimental conditions for the VR-CPT, each providing social cues of different degrees preceding the appearance of the to-be-detected stimuli in a virtual classroom environment so that the cues may direct the participants’ attention toward the stimuli beforehand (Figure 1). In the *control condition*, participants were instructed to complete the two-button AX-CPT, with letters appearing on a white screen in the center, without a virtual classroom environment or a virtual teacher present. This task is essentially equivalent to the traditional computerized CPT, with the exception of wearing an HMD for the baseline measurement of head movements. In the other three VR conditions, the participants were presented with an image of the upper body and face of a male teacher avatar in a virtual classroom setting and asked to complete the CPTs that appeared on the screen to the left or right of the avatar teacher. The three VR conditions differed in how the participants were directed toward the screens to the right or left of the teacher avatar. In the first VR condition (*no-cue condition)*, participants conducted the task without any cues from the virtual teacher; this condition was implemented to determine the effects of social cues from the presence of the avatar teacher alone. Half of the trials were presented on the left screen and the remaining half on the right screen so that the user would have to turn their head once between the two screens. Whether the stimulus was first presented on the right or left screen was counterbalanced across participants according to the program’s protocol.

In the second VR condition (*visual cue condition)*, the teacher avatar chose the left or right screen and directed the participants to look at the screen by pointing. In the third VR condition (*visual/audio cue condition)*, the teacher avatar both pointed and provided a verbal (audio) cue, saying “Look at me,” when he wanted the participant to attend to him (i.e., in between stimuli), and “Look at this,” as he pointed to each screen. The selected screens were changed eleven times under these two conditions. The number of trials appearing on each side of the screen was equal and presented in a counterbalanced order across participants, according to the program’s protocol. The other task variables were equally controlled for throughout all four conditions.

### Dependent measures

#### Head movement data

Head telemetry was measured in degrees (°) of movement in three rotation axes – yaw (Y, horizontal, head turns), pitch (P, vertical, head nods), and roll (R, tilt, lateral head inclinations), and movement was calculated by subtracting the current frame values of every 1/60 s from the previous frame values (i.e., *Y*_*t*_−*Y*_t−1_, *P*_*t*_−*P*_t−1_,*R*_*t*_−*R*_t−1_). The participants needed to turn from screen to screen 11 times in the visual and visual/audio social cue conditions, but required only a single head turn in the no-cue condition, whereas the control condition required no head turns. To eliminate this variability, we marked all timestamps in a log file and excluded yaw movements that occurred at the timestamp of the stimuli presented. The following calculations were performed using these values:

The absolute values of movement (△*Y*,△*P*,△*R*) were indexed by dividing each by the total head movements of that time frame (*THM*_*t*_), defined as the sum of the absolute values of movement for all three axes (e.g., *THM*_*t*_ = △*Y*_*t*_ + △*P*_*t*_ + △*R*_*t*_). The sum of these values was defined as the total magnitude of head movement in each axis during each VR session.

$$Y\,a\,w\,\left(Y\right)=\sum \frac{△\,Y_{t}}{T\,H\,M}$$

$$P\,i\,t\,c\,h\,\left(P\right)=\sum \frac{△\,P_{t}}{T\,H\,M}$$

$$R\,o\,l\,l\,\left(R\right)=\sum \frac{△\,R_{t}}{T\,H\,M}$$

In addition, considering that only horizontal movement would be needed to direct one’s point of view from one screen to another, the sum of the vertical and tilt movements in each time frame was calculated to represent the *task-irrelevant head movement* of the time frame and again summed up as the task-irrelevant head movement during the entire task.

$$T\,a\,s\,k-i\,r\,r\,e\,l\,e\,v\,a\,n\,t\,h\,e\,a\,d\,m\,o\,v\,e\,m\,e\,n\,t=\sum \frac{△\,P_{t}+△\,R_{t}}{T\,H\,M}$$

The VR system for the VR-CPT consisted of a desktop computer with the Microsoft Windows 7 operating system, a high-end graphics card (NVIDIA GeForce GTX 970) with 16GB memory, a monitor, keyboard, speaker, and an Oculus Rift Stereo-HMD with a tracker (DK2 HMD; Oculus lift, Oculus VR, Irvine, CA), and a screen resolution of 960 × 1080 pixels per eye. The VR-CPT was designed using a commercial three-dimensional development platform (Vizard 5.1; WorldViz, Santa Barbara, CA, United States).

### Statistical analysis

For ADHD-RS and CBCL, data with values higher than 1.5 interquartile ranges above the 3rd quartile or lower than 1.5 interquartile ranges below the 1st quartile were excluded as outliers. One participant from the ADHD group had abnormally measured head movement data and was excluded in analyses regarding the data. Independent sample *t*-tests and chi-square tests were used to compare the ADHD and HC groups in terms of demographics, clinical characteristics, and attention variables. Because the ADHD-RS total score and CBCL attention problems subscale did not follow normal distribution when appropriate outliers were excluded, a Spearman’s *r* bivariate correlation test was conducted to analyze the associations between head movement data and ADHD symptom severity. Most head movement data satisfied normality via the Shapiro-Wilk test and the Q-Q plots were close to the line, and thus linear mixed models were constructed to compare differences in task-irrelevant head movements according to the four conditions, both within each group and between the groups. Post-hoc analyses were corrected using the false discovery rate (FDR) correction method. SPSS for Windows (version 23.0; SPSS, Chicago, IL, United States) was used for the *t*-tests, chi-square tests, and the correlation analysis and the SAS version 9.4 (SAS Institute, Cary, NC, United States) was used for the development of linear mixed models. All analyses were considered statistically significant if two-tailed *p* < 0.05.

## Results

### Participant demographics and clinical characteristics

The demographic and clinical characteristics of the ADHD and HC participants were compared using independent *t*-tests, as shown in Table 1. For accurate comparison, outliers were excluded from analyses of simulator sickness questionnaire results, ADHD-RS scores, and CBCL attention problem scores, as few of the participants showed high scores in the extreme for these categories. There were no significant differences in the mean age, IQ, or sex ratio between the two groups. As expected, the two groups had statistically significant differences in ADHD-RS scores [*t* (35) = 4.56, *p* < 0.001] and CBCL attention problem subscale scores [*t* (34) = 2.18, *p* = 0.016].

**TABLE 1:** Demographic and clinical characteristics.

	ADHD (*n* = 20)	HC (*n* = 17)	
		
*Variables*	*M*(SD)	*M*(SD)	*p*-value
Sex, male (%)	90.0	83.3	0.544
Age (years)	11.85 (2.74)	12.12 (2.39)	0.756
Intelligence quotient	104.39 (9.64)	108.65 (9.85)	0.205
Presence questionnaire (PQ)	141.65 (26.94)	153.88 (33.25)	0.225
Simulator sickness questionnaire (SSQ)	6.90 (8.71)	5.56 (8.12)^a^	0.685
ADHD-RS total score	21.79 (11.77)^b^	5.94 (4.45)	<0.001**
CBCL attention problem subscale	60.50 (9.12)^c^	54.18 (4.95)	0.016*

^a^n = 16 for the HC group, ^b^n = 19 for the ADHD group, ^c^n = 18 for the ADHD group, *p < 0.05, **p < 0.001.

### Correlation analysis between head movements during VR-CPT and ADHD symptom severity

**FIGURE 2:**
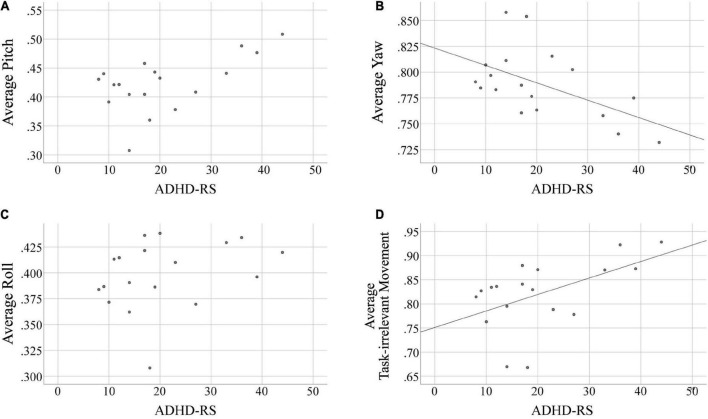
Correlation analyses in the ADHD group between head movement measurements and the ADHD-RS total score. **(A)** Correlation with average pitch, **(B)** correlation with average yaw, **(C)** correlation with average roll, **(D)** correlation with average task-irrelevant movement. ADHD-RS total scores of the ADHD group had statistically significant correlations with yaw movement (i.e., task-relevant head movement) and task-irrelevant head movement averaged across all four conditions. Linear fit lines are provided for results with *p* < 0.05.

Spearman’s correlation tests were used to examine the association between head movement measurements and scores from the ADHD questionnaire results (Table 2). Head movement data were averaged across all four conditions. One participant’s ADHD-RS total score and two participant’s CBCL subscale score was emitted from the ADHD subgroup due to abnormally high results. One additional participant was excluded from the ADHD group because of abnormally measured head movement data. In the ADHD group, there were significant correlations between average yaw (i.e., the average of relatively task-relevant head movement, *r* = –0.492, *p* = 0.038), average task-irrelevant head movement (*r* = 0.489, *p* = 0.040), and the summary index of ADHD-RS in the ADHD group (Figure 2), while no such correlation was observed in the HC group. No significant correlations were found with the CBCL attention problems subscale for both groups.

**TABLE 2:** Correlation analyses between head movement measurements averaged across all conditions, ADHD-RS total score.

*Variables*		ADHD [*r* (*p*-value)]	HC [*r* (*p*-value)]
ADHD-RS total score^a^	Average yaw	–0.492 (0.038*)	-0.141 (0.590)
	Average pitch	0.459 (0.056)	0.263 (0.308)
	Average roll	0.353 (0.150)	0.263 (0.308)
	Average task-irrelevant movement	0.489 (0.040*)	0.239 (0.355)

^a^n = 18 for the ADHD group, n = 17 for the HC group, *p < 0.05.

### Comparison of head movement in the setting of different social cue conditions

To investigate whether social cues presented from a teacher avatar affect the head movement and task performance of participants in a differential manner during the VR-CPT, linear mixed models were constructed for each group with each VR condition as the condition factor and task-irrelevant head movement as dependent variables. One participant from the ADHD group was excluded because of abnormally measured head movement data (ADHD group, *n* = 19; HC group, *n* = 17). Chronological age and IQ were included in the analyses as covariates to disentangle their effects on the participants’ performance. Age was specifically included as a covariant because a strong correlation was found between age and task-irrelevant movement in the ADHD group.

We found a significant main effect of condition (*F*_34_ = 0.10, *p* < 0.001) and group-by-condition interactions (*F*_34_ = 223.84, *p* = 0.031), but no significant effect of group (*F*_34_ = 3.33, *p* = 0.752). Post-hoc analyses with FDR correction are shown in detail in Table 3. There were significant differences between all conditions (all *p* < 0.001, except between the visual and visual/audio cue conditions in the HC group, *p* = 0.016), apart from showing no significant difference between the visual and visual/audio cue conditions in the ADHD group (*p* = 0.615). When corrected for multiple analyses, there was no significant difference observed in the post-hoc analysis of group-by-condition interactions (Table 4).

**TABLE 3:** Post-hoc analysis results for the linear mixed model on the main effect of condition.

	ADHD^a^		HC^b^	
	
Conditions	Effect size	*p*-value (FDR)	Effect size	*p*-value (FDR)
Control vs No-cue	2.144	<0.0001**	2.627	<0.0001**
Control vs Visual cue	4.547	<0.0001**	4.255	<0.0001**
Control vs Visual/audio cue	4.307	<0.0001**	5.107	<0.0001**
No-cue vs Visual cue	2.400	<0.0001**	2.084	<0.0001**
No-cue vs Visual/Audio cue	2.258	<0.0001**	2.834	<0.0001**
Visual cue vs Visual/Audio cue	0.069	0.6147	0.471	0.0164*

^a^n = 19, ^b^n = 17, *p < 0.05, **p < 0.0001.

**TABLE 4:** Post-hoc analysis and Bayesian factor computation results for the post-hoc analyses on the group-by-condition interactions of the linear mixed model.

ADHD vs HC	FDR correction	Bayesian factor	Interpretation
Control vs No-cue	0.3684	0.655	Anecdotal evidence for H0
Control vs Visual cue	0.5781	0.376	Anecdotal evidence for H0
Control vs Visual/audio cue	0.5781	0.406	Anecdotal evidence for H0
No-cue vs Visual cue	0.0888	3.727	Moderate evidence for H1
No-cue vs Visual/audio cue	0.5781	0.364	Anecdotal evidence for H0
Visual cue vs Visual/audio cue	0.1082	1.942	Anecdotal evidence for H1

H0: null hypothesis (i.e., the magnitude of difference in task-irrelevant head movement between the two conditions are not different in the two groups).

H1: alternative hypothesis (i.e., the magnitude of difference in task-irrelevant head movement between the two conditions are different in the two groups).

Bayesian factors were computed for each insignificant result. For the main effect of condition between the visual and visual/audio cue conditions in the ADHD group, the computed Bayesian factor was 0.321, indicating presence of moderate evidence supporting the null hypothesis (i.e., there is no difference in task-irrelevant head movement between the two conditions in the ADHD group). For group-by-condition interactions, the Bayesian factors are presented in Table 4. Most interactions had anecdotal evidence for either the null or the alternative hypothesis, indicating that results are largely insignificant.

**FIGURE 3:**
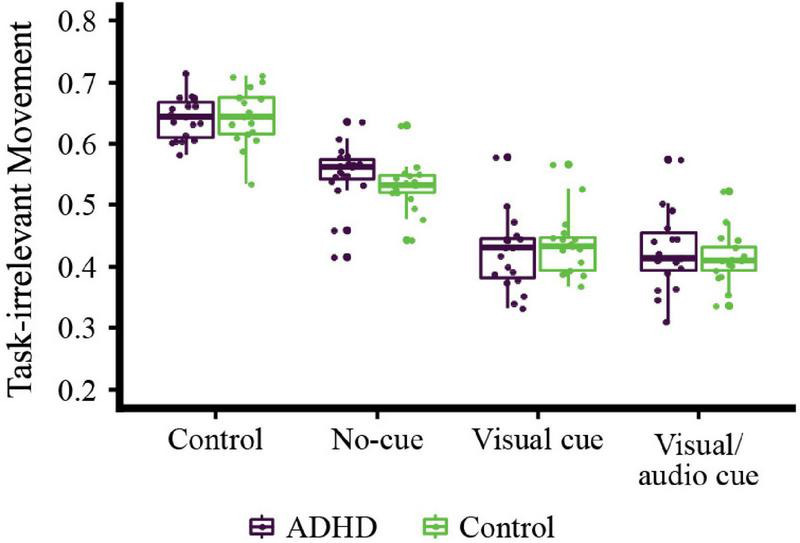
Comparison of task-irrelevant head movement between conditions by group. The main effects of the condition and group-by-condition interactions were statistically significant, while the main effects of group were not. According to the post-hoc analyses performed with FDR correction, the healthy control (HC) group showed statistically significant differences between all conditions; however, the ADHD group did not show a significant difference of task-irrelevant head movement between the visual and visual/audio cue conditions.

Therefore, in terms of the overall trend, there was a decreasing trend in task-irrelevant head movement in the presence of the virtual classroom environment and again with visual and/or audio cues added (Figure 3). However, the magnitude of difference in task-irrelevant movement according to changes of condition was not different in the two groups.

## Discussion

Our study examined the impact of different contexts simulated within a VR classroom on the head movements of children and adolescents with and without ADHD. Task-irrelevant head movements in the ADHD group showed a significant correlation with the current standard ADHD clinical scale. In addition, by utilizing digital technology to strategically alter various components, the participants were exposed to different sensory modalities of social cues. The head movement of children with ADHD seemed to decrease in response to a virtual avatar teacher and their social cues compared to when they were absent. While visual cues seemed to positively influence the attention of both ADHD and HC individuals, the effect of an additional audio cue is debatable. Overall, these results imply that virtual environments provide researchers with a platform on which rigorous experimental control and manipulation can be applied while simultaneously enabling the collection of objective behavioral data. Our results also demonstrate that the alteration of physical or social components in VR may be used as an effective method for optimizing students’ attention.

### Relationship between head movement during virtual reality-continuous performance test and attention deficit hyperactivity disorder symptom severity

Our results showed a significant positive correlation between task-irrelevant head movement during the VR-CPT with visual/audio social cues and ADHD-RS scores, the most commonly used standard scale to measure ADHD symptoms in clinical practice. This result supports our first hypothesis that head movement data in a virtual environment can effectively represent the degree of ADHD symptoms, reflecting real-life performance reported via ADHD-RS scales. This correlation was not observed in the HC group, possibly implying the specificity of this mode of measurement in the ADHD group. However, these results should be interpreted with our small sample size in consideration. Future replicative studies with a larger sample size would be needed to solidify our findings.

Discovering and defining objective means to quantify the symptoms of ADHD is important, as it may compensate for the lack of objectivity in the current method, which relies on self-or parent-reported ADHD symptom scales. This finding adds to the small but growing body of literature utilizing automated measurement of body and head movement as one such method. However, in our study, no significant differences were observed in task-irrelevant movements between the two groups. This result is inconsistent with previously published meta-analytic results, indicating a higher level of movement in children with ADHD (Parsons et al., 2007; Diaz-Orueta et al., 2014; Nolin et al., 2016). The reason for these inconsistent results is unclear, but may be because VR generally enhances the attention level of children with ADHD through motivation and entertainment (Rizzo et al., 2004; Dovis et al., 2012; Larson et al., 2014), and the inclusion or exclusion of distractions causes or diminishes performance differences between ADHD and HC participants (Mansfield et al., 2003; Rizzo et al., 2004; Rizzo et al., 2006; Mazurek and Engelhardt, 2013; Eom et al., 2019). Our VR setting was relatively simple without significant distractions, in addition to an HMD that blocked distractions from the outside real world (Rizzo et al., 2006; Bailenson et al., 2008), and thus, may have been an ideal environment for ADHD participants to concentrate on the task more easily. The different objective measures used in the assessment of hyperactivity may, in part, explain the inconsistency of the results among studies. Perhaps more sophisticated activity indicators will be more effective in discriminating between the two groups. Other objective locomotion measures, such as actigraphy and infrared motion tracking systems with computerized attention testing, have been shown to be useful for evaluating inattention and hyperactivity/impulsivity symptoms in children and adults with ADHD (Teicher et al., 1996; García Murillo et al., 2015; Hall et al., 2015; Lee et al., 2018). Regardless, our setting of the VR environment was sufficient to analyze the patterns of head movement in both groups, reflecting their real-life performance reported via the ADHD-RS scales.

In addition, the VR classroom environment itself seemed to decrease the hyperactivity of ADHD and HC participants, as reflected by the decrease in the task-irrelevant head movement of participants during the no-cue condition compared to the control condition. VR-CPT has been seen to captivate the attention of children with ADHD more effectively than traditional CPTs in many studies (Rizzo et al., 2004; Dovis et al., 2012; Mazurek and Engelhardt, 2013), as well as having a motivating effect on children (Rizzo et al., 2004; Dovis et al., 2012; Larson et al., 2014). Participants with ADHD often report VR-CPTs as being more engageable than the conventional 2D computerized version (Dovis et al., 2012; Larson et al., 2014). Previous studies have also implied that students can learn more by being in the center of a teacher’s field of view rather than in the periphery by being closer to the teacher (Bailenson et al., 2008). Because our VR setting placed the teacher avatar directly in front of the participant’s point of focus, it may have generated the effect of being in the place of a student sitting in the middle of the front row of a classroom, in the proximity of the teacher. This finding suggests that VR environments have the potential to alter the physical dynamics of learning environments and can be utilized to better treat or educate patients with ADHD.

### Effect of the teacher avatar’s cues on the attention of attention deficit hyperactivity disorder and healthy control participants

We explored whether head movement varied depending on the addition of visual cues only and both visual and audio cues provided by a teacher avatar and found that head movement could be affected by these social cues in a virtual classroom environment. In this study, we found no significant group differences in task-relevant or task-irrelevant head movement between children with ADHD and HCs; however, significant differences across conditions were found, which we attributed to the different VR conditions.

Regardless of ADHD diagnosis, previous studies have revealed that social cues tend to improve students’ attention performance. For example, in a classroom environment, the synchronous behavior of the teacher and students can increase rapport and result in better learning outcomes (Zhou, 2012; Gaastra et al., 2016). Coherent results were found in a study investigating the movements of teachers and students using machine learning algorithms, which showed that synchronous nonverbal behavior led to increased attention and higher learning scores (Won et al., 2014). Taken together, the cues of the teacher avatar in our VR classroom environment may have contributed to the attention level of participants by promoting them to carry out head movements synchronous to the teacher’s guidance provided by gestural and vocal cues.

Group-by-condition interaction for the pairs of conditions were not statistically significant, against our third hypothesis that the two groups would respond differently to the two conditions. However, as we have stated in section 4.1, significant correlation between task-irrelevant head movement and ADHD-RS was only observed in the ADHD group, and not in the HC group, possibly indicating that task-irrelevant head movement may represent clinically significant hyperactivity/inattention in those with ADHD diagnosis only. Therefore, it may be questionable to say that this represents clinically significantly increased attention for the HC group, and the interpretation of the group-by-condition interaction should be done with care. Here, we focused on the difference in task-irrelevant head movement by condition in the ADHD group and propose some theories based on previous studies that may provide a reasonable explanation for why there was no significant decrease in the amount of task-irrelevant head movement when audio cues were added to the visual cue condition in the ADHD group.

First, it is possible that the results were influenced by the poor multisensory integration ability of the ADHD participants. While synchronous auditory stimuli may act synergistically with visual stimuli by increasing connectivity between the low-level visual and auditory areas of the brain (Romei et al., 2009; Lewis and Noppeney, 2010; Beer et al., 2011), the synchronicity itself is based on the integrity of the multisensory integration process influenced by an individual’s attention level. Children and adolescents with ADHD who are attention-deficient by definition may have difficulties in integrating multisensory stimuli (i.e., visual and audio cues simultaneously) to search for stimuli requiring attentional focus (Romei et al., 2009; Tang et al., 2016).

Our second theory is based on the use of top-down (i.e., endogenous) and bottom-up (i.e., exogenous) orientation in head movement (Frischen et al., 2007). Bottom-up orientation refers to external stimuli in one direction leading to an unconscious draw of attention toward that direction. In contrast, top-down orientation is a goal-driven process that is triggered by one’s selection to focus on a certain stimulus. While the teacher’s raised forearm in one direction likely triggered some bottom-up orientation, the audio (verbal) cue in our study was provided by the teacher in the center of the two screens, lacking any localization effect. This may have driven the receiver to interpret what the sound means and determine focus. Thus, the results of our study imply that children with ADHD effectively utilize bottom-up orientation but cannot utilize top-down orientation at the same time to improve their focus, consistent with reports from previous studies (Egeland et al., 2010; Petrovic and Castellanos, 2016).

Lastly, while it was our intention for both visual and visual/audio cues to be perceived as social cues, it is possible that the visual cue may have acted as a simple “pointer” and hence had partial properties as a non-social cue (Frischen et al., 2007; Seernani et al., 2021), unlike the verbal cues given from the center of the visual field. In the presentation of valid and invalid cues prior to stimuli in spatial cueing paradigms, children with ADHD have been observed to be able to use non-social cues, but not social cues, to orient their attention (Marotta et al., 2014). The disproportionately impaired orienting ability of children with ADHD may explain how the presence of only visual cues could cause significant changes in task-irrelevant head movements.

To conclude, it is unclear within our study which theory provides the best explanation for the absence of head movement difference between the visual vs visual/audio cue conditions in the ADHD group. Revealing the actual reason behind our results and clarifying whether the two groups are different in each condition would require additional investigation, including assessment of other body movements (Teicher et al., 1996; García Murillo et al., 2015; Hall et al., 2015; Lee et al., 2018) and/or addressing whether the presence of the two modalities together would have a larger impact on the attention performance and body movements of children with ADHD compared to each single modality (visual or audio).

### Limitations

This study has several limitations. First, our VR was designed to be relatively simple, and may not demonstrate ADHD-related differences to the same extent as in real-life situations. More work is needed to better understand how attention performance is influenced in these simulated situations compared to an individual’s behavior in real life.

Second, the absence of distractions, which inevitably exist in a real classroom environment, is an important limitation. Deeper insight into the underlying mechanism of how non-social and social stimuli affect the attention-orienting process of ADHD may be obtainable if a more sophisticated distractor setting is created. Third, post-hoc power analysis for our study revealed limited power, implying that our sample size may not have been large enough to provide substantial experimental power. Also, the male-to-female ratio was high, which may be a limitation in generalizing our results to female patients. Fourth, even though participants in the ADHD group were asked to withhold methylphenidate 48 h prior to the study, its effect cannot be completely ruled out. Although it would have been preferable to require children to participate after a longer period of medication abstinence, this may have exposed our study participants to an unnecessary burden. Additionally, the comorbidities included in our inclusion criteria may have influenced the results. Fifth, while previous exposure to video games and VR (Maneuvrier et al., 2020), or self-representation of oneself as an avatar (Friehs et al., 2022) may influence CPT performance within VR environments, we did not control for these factors in our study.

Although the use of VR improves the ecological validity of attention task performance, our study setting was still based on the traditional CPT format, making it difficult to represent real-life attentional capacity (Parsons, 2015). Further research with a novel task that better reflects real-life classroom environments is necessary to overcome this limitation. Another useful future investigation would be the analysis of eye movement using an eye-tracking device, given that participants may have moved their eyes without moving their heads to carry out the CPT task (Lev et al., 2020). However, the use of head-mounted eye trackers may pose practical difficulties, including low tolerance of children to these devices, which may be even more troublesome for individuals with ADHD. Finally, pitch, yaw, and roll were moderately correlated in our data, although they were examined separately in our study. A possible alternative approach would be to include body movements to reflect hyperactivity more precisely.

## Conclusion

We quantified head movements in children and adolescents with and without ADHD using automated measurement, which showed different tendencies depending on the presence or absence of social and non-social stimuli. These findings suggest the possibility of using automated head motion measurement in a VR environment as a reliable objective method to quantify the hyperactivity component of ADHD symptoms to supplement the current subjective manner of ADHD symptom measurement. This proof-of-concept study not only provides a new, relatively ecologically valid way of conducting studies aimed at understanding how various cues affect the attention of children in a classroom environment, but may also improve the understanding of nonverbal behavior in ADHD and assist with designing online educational systems for those affected. Further research may reveal a more extensive potential of VR technology in identifying and longitudinally monitoring ADHD symptoms, as well as providing a clinical aid as a digital phenotype to stratify severity.

## Data availability statement

The raw data supporting the conclusions of this article will be made available by the authors, without undue reservation.

## Ethics statement

The studies involving human participants were reviewed and approved by Institutional Review Board of Yonsei University College of Medicine, Gangnam Severance Hospital. Written informed consent to participate in this study was provided by the participants or their legal guardian/next of kin.

## Author contributions

EK and KK designed this study. HE and YH collected the data. JYY, YJC, and HSL performed statistical analyses. JYY and YJC wrote the manuscript. EK, JYY, JK, HSL, and BS contributed to the discussion and reviewed and edited the manuscript. All authors have read and approved the final version of the manuscript.
